# Cerebrospinal fluid biomarkers of malfunctioning ventriculoperitoneal shunts: a pilot study

**DOI:** 10.1186/2045-8118-12-S1-O20

**Published:** 2015-09-18

**Authors:** Clinton David Morgan, Diego M Morales, Gakwaya Habiyaremye, James P McAllister, David D Limbrick

**Affiliations:** 1 Department of Neurological Surgery, Vanderbilt University School of Medicine, Nashville, TN, USA; 2 Department of Neurological Surgery, Division of Pediatric Neurosurgery, Washington University, St. Louis, MO, USA

## Introduction

Ventriculoperitoneal shunts (VPS) are a critical feature of management of pediatric hydrocephalus. Unfortunately, shunt failure is often unrecognized before significant symptoms and focal signs present, requiring costly emergency room visits and neuroimaging. Ventricular CSF biomarkers of neuropathology and neurodevelopment are emerging as critical predictors of real-time physiology in pediatric hydrocephalus. Moreover, in the future, biomarkers of neuropathology could be pre-operatively analyzed from shunts in question, reducing unnecessary surgical exploration. In this pilot study, CSF samples taken during surgical exploration were compared, hypothesizing that that two critical CSF proteins, amyloid precursor protein (APP) and neural cell adhesion molecule (NCAM-1), known to be elevated in untreated hydrocephalus, may discriminate between functioning and malfunctioning shunts.

## Methods

Approval from the Washington University Human Research Protection Office was acquired, and a pediatric CSF repository was established. All patients prospectively enrolled were between 0-4 years of age and were undergoing surgical exploration of proximal, valve, and distal components of VPS for suspected CSF diversion failure. CSF was acquired in the operating room under sterile conditions, transported on ice, centrifuged, and supernatant was stored at -80°C until experimental analysis. Enzyme-linked immunosorbent assay (ELISA) was used to examine CSF concentration of APP and NCAM-1. Total protein was assessed using a standard Pierce Bicinchoninic Acid (BCA) protein assay kit. A microplate reader was used to assess biomarker concentration (ng/mL). Mann-Whitney U analysis was used for comparison of these non-parametric data, with statistical significance threshold set a priori at p<0.05.

## Results

Twenty eight patients were found to have malfunctioned VPS, while 5 were found to have functioning VPS. There were no significant differences in age at the time of ventricle CSF sample (p=0.07), levels of total CSF protein (p=0.335), total APP (p=0.516), or total NCAM (p=0.816) concentrations. However, due to the fact that there is significant inter-individual variation in CSF total protein levels, we compared normalized APP (nAPP) and normalized NCAM-1 (nNCAM) between the two groups.

Patients found to have nonfunctioning shunts had a significantly elevated nAPP (p=0.044) and tended to have elevated nNCAM (p=0.061) when compared to patients with functioning shunts.

## Conclusions

nAPP and nNCAM concentrations in ventricular CSF may be elevated in malfunctioning VPS, when compared to similarly aged patients with functioning shunts. While this is a small pilot study, active enrollment continues of new patients into this study given its potential implications.

